# An activity-integrated strategy of the identification, screening and determination of potential neuraminidase inhibitors from Radix *Scutellariae*

**DOI:** 10.1371/journal.pone.0175751

**Published:** 2017-05-09

**Authors:** Wei Liu, Huilin Wang, Bo Zhu, Chengqian Yin, Shuyang Chen, Jin Li, Xie-an Yu, John Teye Azietaku, Mingrui An, Xiu-mei Gao, Yan-xu Chang

**Affiliations:** 1 Tianjin State Key Laboratory of Modern Chinese Medicine, Tianjin University of Traditional Chinese Medicine, Tianjin, China; 2 Tianjin Key Laboratory of Phytochemistry and Pharmaceutical Analysis, Tianjin University of Traditional Chinese Medicine, Tianjin, China; 3 Department of Pharmacology and Experimental Therapeutics, Boston University School of Medicine, Boston, Massachusetts, United States of America; 4 Department of Surgery, University of Michigan, Ann Arbor, Michigan, United States of America; Macau University of Science and Technology, MACAO

## Abstract

Small molecules isolated from herbal medicines (HMs) were identified as the potential neuraminidase inhibitors which are effective in influenza prevention and treatment. Unfortunately, current available screen methods of small molecules isolated from HMs are inefficient and insensitive. Here a novel Ultra Performance Liquid Chromatography coupled with diode-array detectors and auto-fraction collector / time-of-flight mass spectrometry (UPLC-DAD-FC/Q-TOF-MS) screening method with high efficiency was developed and validated to separate, collect, enrich, identify and quantify potential neuraminidase inhibitors from Radix *Scutellariae*. The results showed that 26 components with neuraminidase inhibitory activity were identified from Radix *Scutellariae* extracts. It was also found that the influence of origins on the quality of RS was more than that of cultivated time on the basis of the concentration of the effective components. These results brought novel insights into quality evaluation of *Radix Scutellariae*. It was demonstrated that new activity-integrated strategy was a suitable technique for the identification, screening and determination of potential neuraminidase inhibitors in herbal medicine and will provide novel potential strategies in other drug screening from herbal medicine.

## Introduction

Influenza is a burgeoning infectious disease which causes severe respiratory illnesses [1]. The viral surface protein neuraminidase (NA) is widely used as a direct target to inhibit viral replication and block human body infection [2], which promotes the hydrolysis of terminal sialic acid residues of newly generated virus and host cell receptors [3]. Influenza neuraminidase inhibitors, such as Oseltamivir, Peramivir, Zanamivir, have been widely used for influenza type-A and type-B virus treatment [4,5], but their applications are limited due to drug resistance and side effects including vomiting, nausea, and diarrhea [6]. Therefore, natural neuraminidase inhibitors have attracted increasing attention on the treatment of influenza [7].

It was reported that more than four hundred different kinds of herbal medicines (HMs) has been detected to have high efficiency of NA inhibitory activities [8]. Many of these HMs have been used to treat the Influenza for thousands of years in clinics in China. They are also regarded as an important source of novel pharmacologically active compounds and can be used to identify and purify of natural neuraminidase inhibitors in drug discovery and pharmaceutical research [9]. HMs was the complicated mixture of components[9]. It is therefore hard to screen and identify the effective functional components in HMs. It was very necessary to select some effective components as combinatorial markers for the quality control of these HMs in order to guarantee efficacy and safety. There are many combination of chemical analysis and biological tests methods reported by scholars with different features in order to solvent the quality problem of HMs [11–13], which have overcome to some extent the quality problems in HMs and screening active compounds of complex extracts as markers to evaluate quality of HMs. However, there were no chemical and biological analysis combined methods for rapid screening neuraminidase inhibitors from HMs in the literatures. The current bioactivity—guided fractionation and isolation method was mainly used to screen natural neuraminidase inhibitors as markers for quality control of HM. However, it is so time-consuming, labor-intensive and expensive that screening the effective functional components was challenge in HMs. Recently, Ultra Performance Liquid Chromatography coupled with diode-array detectors and auto-fraction collector / time-of-flight mass spectrometry (UPLC-DAD-FC/Q-TOF-MS) coupled with bioactive assays were used to screen and identify anti-diabetic ingredients from HMs in our previous studies [14]. In light of these, UPLC-DAD-FC/Q-TOF-MS method was here proposed to screen natural neuraminidase inhibitors as combinatorial markers for the quality control of HMs.

Radix *Scutellariae* (Huangqin, RS), the dried root of *Scutellariae baicalensis* Georgi, has been used as one of HMs to treat diseases for thousands of years for its anti-febrile and detoxification functions. Pharmacology studies have demonstrated that Radix *Scutellariae* treatment is effective in anti-inflammatory [10], anti-oxidant [11], anti-cancer [12], anti-angiogenesis [13] and anti-viral activities [1,14]. Photochemistry studies showed that baicalin, wogonoside, baicalein, wogonin and oroxylin A were the major bioactive components in Radix *Scutellariae* [15]. Although RS is considered a potential HM for screening bioactive components of neuraminidase inhibitors, the current available screening and analysis methods are inefficient and insensitive. The inhibitory effects of baicalin and baicalein on neuramidase have been reported based on immobilized NA micro-reactor [16], but it only could be used to test NA inhibitory activities of ingredients or extracts of TCMs, but not rapidly screen the NA inhibitory activities of each component from the HMs extracts.

**Fig 1:**
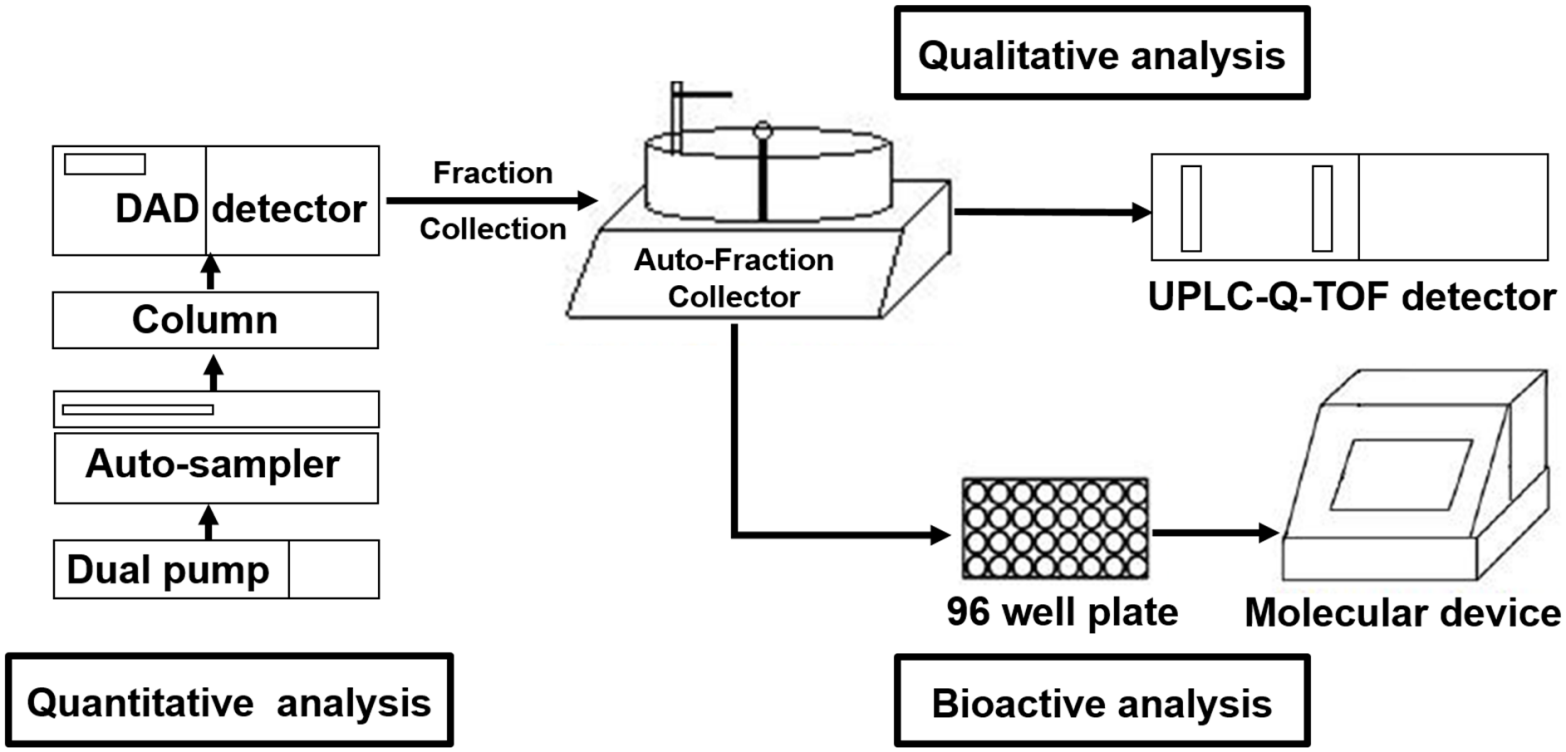
The principle of an activity integrated strategy method. The UPLC system with auto-fraction was used to separate, quantify collect and enrich the active compounds in fraction were performed by an. The fractions were used to test the bioactive analysis.

To our knowledge, UPLC-DAD-FC/Q-TOF-MS application for screening natural neuraminidase inhibitors from RS has not been reported in the literatures. In this study, UPLC-DAD-FC was used to separate, quantify, collect and enrich more components in the fractions of RS. Then neuraminidase inhibitory activities of all fractions were performed. Finally, the UPLC-DAD-Q-TOF-MS system was used to identify the components in fractions with neuraminidase inhibitory activities from RS (Fig 1). Given the importance of the natural neuraminidase inhibitors, hierarchical cluster analysis was used to distinguish quality control of the different RS samples from different areas basing on their contents. The new activity-integrated strategy of the identification, screening and determination of potential neuraminidase inhibitors in one-step work will become the advantageous tool for screening natural neuraminidase inhibitors in drug discovery and evaluating the quality of herbal medicines for treating Influenza.

## Materials and methods

### Plant preparation and chemicals

The RS samples used were collected from original production base of medical material in China (Table 1). The species were authenticated by Prof. Lin Ma and the voucher specimens were deposited at the Tianjin University of Traditional Chinese Medicine, Tianjin, China.

**Table 1:** The information of the 18 batches of Radix *scutellariae*.

Name	Place of origin	Growth Ages
S1	Planted in Cheng De, He Bei, China	2
S2	Planted in Cheng De, He Bei, China	3
S3	Planted in Cheng De, He Bei, China	4
S4	Planted in Cheng De, He Bei, China	5
S5	Planted in Cheng De, He Bei, China	7
S6	Planted inYun Cheng, Shan Xi, China	3
S7	Planted inYun Cheng, Shan Xi, China	3
S8	Planted inYun Cheng, Shan Xi, China	2
S9	Planted inYun Cheng, Shan Xi, China	2
S10	Planted in Ri Zhao, Shang Dong, China	2
S11	Planted in Ri Zhao, Shang Dong, China	2
S12	Planted in Ri Zhao, Shang Dong, China	1
S13	Planted in Ri Zhao, Shang Dong, China	1
S14	Planted in Ri Zhao, Shang Dong, China	2
S15	Luan Ping, He Bei, China of wild	/
S16	Wu An, He Bei, China of wild	/
S17	Fu Ping, He Bei, China of wild	/
S18	Qing Hai, China of wild	/

Acetonitrile was purchased from Dikma Technologies Inc. (USA) and methanol was obtained from Tianjin Concord Science Co. Ltd. (Tianjin, China). Formic acid was obtained from Tianjin Kermel Science Company. Deionized water was processed with the Milli-Q Academic ultra-pure water system (Milliporein, USA). The neuraminidase inhibitors screen kit was purchased from Beyotime (Shanghai, China). Reference standards of scutellarin, scutellarein, baicalein, baicalin, wogonoside, wogonin, chrysin-7-O-glucuronide, chrysin and oroxylin A (purity > 98%) were obtained from Chinese National Institute for Control of Pharmaceutical and Biological Products (Beijing, China).

### UPLC-DAD-FC system

Waters ACQUITY UPLC System (Waters Co., Milford, MA) equipped with a diode array detector (DAD) was employed in separating, quantifying and collecting the multiple components in RS sample. The workstation controlled by Empower 2 software was used to collect and analyze data. The ACQUITY UHPLC BEH C18 column (2.1× 100 mm, 1.7 μm) (Waters Co.) was used in separating the various components. The mobile phase comprised 0.1% (v/v) formic acid aqueous solution (A phase) and acetonitrile (B phase) employing a gradient elution of 0–2 min, 10–10% B; 2–2.5 min, 10–14% B; 2.5–9 min, 14–15% B; 9–13 min, 15–16% B; 13–15 min, 16–16% B; 15–20 min, 16–20% B; 20–25 min, 20–32% B; 25–26 min, 32–35% B; 26–35 min, 35–40% B. The flow rate was set at 0.3 mL min^-1^, with a column temperature fixed at 35°C. The injection volume was 1 μL and detection wavelength was 280 nm.

The fraction collection and enrichment of the active compounds were achieved with auto-fraction collector (FC) (BSZ-100, Shanghai Qingpu Huxi Instrument, Shanghai, China) and the UPLC system was employed in separation of the extract from RS and quantitative determination of the natural active components. Using a time span between different peaks in the chromatographic profile of the UPLC and a fixed-time interval collection based on the retention time, the various fractions were collected automatically. Accordingly, a time interval of 21 s was selected to collect the active compounds enriched by the UPLC separation. The samples were subsequently dried using nitrogen gas and the residues were dissolved in methanol for the activity assay.

### UPLC-Q-TOF-MS system

UPLC-Q-TOF-MS system (Agilent Co.) was used to identify the components in RS samples with the ESI-MS spectra obtained in both the negative and positive ion modes. The conditions of the capillary voltage (CV) of the positive ion mode and negative ion mode were +3.5 kV and -3.5 kV, respectively. The nebulization and auxiliary gas was high-purity nitrogen (>0.9999) with pressure of nebulizer gas of 35 psig and the flow rate of drying gas of 8.0 L min^-1^ at temperature of 350°C. Fragmentor voltage (FV) and skimmer voltage (SV) were set at 175 and 65 V, respectively. The collision energy (CE) selected ranged from 15 V to 40 V. Data was collected for MS at MS scan ranging 100–1700 Da and for MS/MS at MS/MS scan ranging 50–1500 Da. The Mass Hunter software (Agilent Technologies) was employed to acquire data including the exact mass and to analyze the elemental compositions. The mobile phase used in UPLC-Q-TOF-MS analysis comprised formic acid aqueous solution (0.1%, v/v) (A phase) and acetonitrile (B phase). The separation condition was identical to that of the UPLC-UV-FC system.

### Preparation of sample solutions

#### RS sample preparation

0.1 g of the RS powder was mixed with 10 mL of 50% methanol (methanol-water) in ultrasonic bath for 30 min. The mixture was centrifuged at 14,000 rpm for 10 min and the supernatant was diluted four times for UPLC system analysis or screening assay.

#### Standard solutions

Standards of scutellarin (0.5 mg mL^-1^), scutellarein (0.5 mg mL^-1^), baicalin (1 mg mL^-1^), baicalein (0.5 mg mL^-1^), wogonoside (0.5 mg mL^-1^), wogonin (0.5 mg mL^-1^), chrysin-7-O-glucuronide (1 mg mL^-1^), chrysin (1 mg mL^-1^) and oroxylin A (1 mg mL^-1^) were respectively prepared in 100% methanol for quantitative determination and 10% methanol for bioassays, and then diluted to the working concentration.

### Calibration curve, repeatability, LODs and LOQs

In constructing the calibration curves, each mixed standard solution was determined in triplicate at the various concentrations diluted with that of the standard stock solution. A plot of the peak areas (y) against the corresponding concentration (x, μg mL^-1^) showed the calibration curve of each standard. The repeatability, expressed as the relative standard deviation (RSD), was validated using six independent samples.

The LOD and the LOQ were defined as the lowest concentration for quantification and detection respectively, obtained by diluting the lowest concentration of the mixed standard solution. The LOD and LOQ were calculated at a signal-to-noise (S/N) ratio of 3 and 10, respectively.

### Precision and stability

Precision was calculated by measuring the intra-day and inter-day variability and expressed as RSD. A preparation of the stock standard solutions at three different concentrations namely low, medium and high concentrations was analyzed and used to calculate the RSD. The mixed standard solutions were tested for six replicates within one day to estimate the intra-day precision. On the other hand, the inter-day variability assay was carried out by examining the mixed standard solutions in replicates for three days consecutively. At time intervals of 0, 2, 4, 6, 8, 12 and 24 h, the method stability was determined with the low, medium and high concentration of the mixed standard solutions by performing six repetitive injections of the samples. The accuracy ranging ±15% of the primitive concentration and RSD less than 5% were considered to be acceptable.

### Recovery

The recovery was tested as follows: spiking 100% quantities of the investigated compounds into a certain amount of the RS sample. Extraction and analysis were performed on the resultant samples using the methods mentioned above. The recovery was calculated by the formula: Recovery (%) = (found amount original amount)/spiked amount × 100%.

### Bioassay of neuraminidase inhibition

Following the manufacture’s instruction, the NA inhibition experiment was carried out in a 96-well microplate reader [17]. In each well, 70 μL of reaction buffer solution, 10 μL of NA and 10 μL of standard samples dissolved by methanol were mixed together. Mixture was vibrated for 1 min and then incubated at 37°C for 2 min. After incubation, 10 μL of fluorescent substrate (2′-(4-Methylumbelliferyl)-α-D-N-acetylneuraminic acid sodium salt hydrate) was added. The mixture was subsequently vibrated for 1 min and then incubated at 37°C for 30 min. The fluorescence intensity was measured by a fluorescence multifunctional microplate reader (Tecan GENios, Männedorf, Switzerland) with excitation at 485 nm and emission detection at 530 nm. Water was used in the control group instead of the standard samples.

The inhibition rate (R%) was calculated by a formula: (A1−A2)/A1×100. A1 is the absorbance of the control and A2 is the absorbance of the sample. The IC_50_ was determined by plotting the percentage of NA activity against inhibitor concentration. All samples were determined in triplicates.

### Cell culture, cell viability and transfection

Normal human primary lung fibroblasts (PLF) (ATCC^®^ PCS201013^™^) were cultured in Fibroblast Basal Medium (ATCC^®^ PCS201030^™^) supplemented with growth factors. HIEC-6 cells were cultured in the complete growth medium according to the protocols as described previously [18,19]. The HEK293T cells were cultured in DMEM medium with 10% FBS. The MTT assay was employed to test cell viability. In brief, cells were seeded into 96-well plates at a density of 1 × 10^5^ cells well^-1^. 24 hours after treatment of indicated drugs, the culture medium was replaced with 5 mg mL^-1^ MTT assay solution (Sigma-Aldrich) and incubated for 4 hours. The reaction was terminated in 150 μL anhydrous dimethyl sulfoxide (DMSO, Sigma-Aldrich). The absorbance at 570 nm was measured with the microplate reader. The viability value was calculated as average absorbance of experimental group/average absorbance of control group.

NA plasmid pNAHA was purchased from addgene (Plasmid #44169)[20], and transfection was performed by using Lipofectamine 3000 Reagent (Life Technologies) according to the manufacturer’s protocol. HEK293T cells transfected with neuraminidase expression plasmid were grown in 24-well plate and treated by NA activity compound for 24h. Then the whole cell extract was used for NA inhibitory assay as mentioned in Bioassay of neuraminidase inhibition.

## Results and discussion

### Optimization of chromatographic conditions

**Fig 2:**
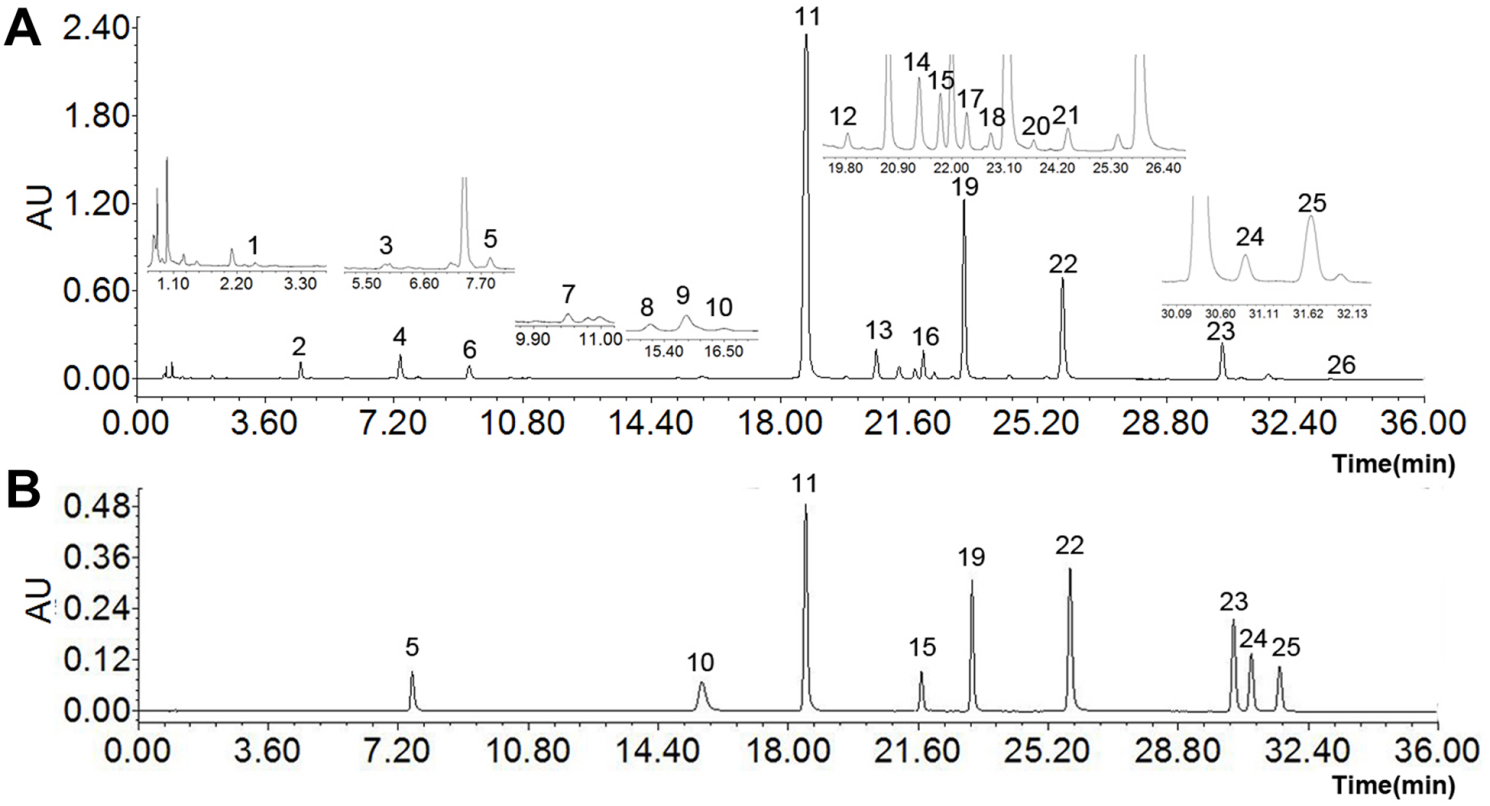
Typical chromatogram. **(A) RS extracts, (B) 9 standards mixture.** Scutellarin (5), scutellarein (10), baicalin (11), chrysin-7-O-glucuronide (15), baicalein (19), wogonoside (22), wogonin (23), chrysin (24) and oroxylin A (25).

To improve peak resolution efficiently, UPLC chromatographic conditions were optimized, including mobile phases (acetonitrile—water, methanol—water), concentrations of formic acid (0.1% and 0.2%), column temperatures (30, 35 and 40°C) and flow set (0.2, 0.3 and 0.4 mL min^-1^). By comparing the shapes of the peaks and the resolutions of the target compounds, the best results were obtained in the conditions with acetonitrile 0.1% formic acid as a mobile phase. The column temperature was 30°C and flow was set at 0.3 mL min^-1^ (Fig 2).

### Method validation

To validate the newly established UPLC method, the total 9 standard markers, including scutellarin, scutellarein, baicalin, baicalein, wogonoside, wogonin, chrysin-7-O-glucuronide, chrysin and oroxylin A, were used. As shown in Table 2, the calibration curves for the 9-targeted standard markers exhibited a good linearity (R^2^> 0.9990). The relative standard deviations (RSDs) of repeatability were lower than 3.00%. The LODs and LOQs of each analyte ranged from 0.13 to 0.25 μg mL^-1^ and 0.25 to 0.50 μg mL^-1^as shown in Table 2, respectively.

**Table 2:** UHPLC data for the calibration curves, LOQs, LODs and repeatability (n = 6).

Compounds	Regressive equation	Linear range (μg mL^−1^)	R^2^	LOD (μg mL^−1^)	LOQ (μg mL^−1^)	Repeatability RSD (%)
Scutellarin	y = 10742x + 4129.8	0.4–100	0.9992	0.25	0.40	1.99
Scutellarein	y = 16046x – 5356.3	0.8–100	0.9990	0.33	0.50	1.35
Baicalin	y = 11911x + 584.09	2.0–500	0.9993	0.13	0.25	1.79
Chrysin-7-O-glucuronide	y = 8648.2x + 1668.5	0.8–100	0.9990	0.13	0.50	1.00
Wogonoside	y = 12743x + 7097.2	1.6–200	0.9990	0.13	0.25	1.17
Baicalein	y = 19801x + 30728	1.6–200	0.9990	0.13	0.25	1.43
Wogonin	y = 24854x + 7714.8	0.8–100	0.9990	0.13	0.25	1.34
Chrysin	y = 15839x – 3236.8	0.8–100	0.9990	0.13	0.50	0.95
Oroxylin A	y = 13130x + 4699	0.8–100	0.9990	0.25	0.40	2.11

The relative standard deviations (RSDs) of intra-day and inter-day precision were 0.17–2.62% and 0.47–2.84%, respectively. The intra-day and inter-day precision for the 9 standards accuracies were 95.0–107% and 92.6–108%. These standards were showed to be stable in 24 h. The relative standard deviations (RSDs) were < 3%. Furthermore, the accuracies ranged of stability was from 94.9% to 107% (Table 3). The relative standard deviations (RSDs) of all recoveries were < 3% with the accuracy ranging of 98.7–104%, except for oroxylin A. Oroxylin A was not separated from skullcapflavone II by UPLC (Table 4). All these results indicated that the precision and stability of the developed method were reliable for the quantitative analysis of the naturally active components in RS.

**Table 3:** Intra-day, inter-day precision and stability of the seven bioactive compounds (n = 6).

Compounds	Concentration (μg mL^−1^)	Inter-day		Intra-day		Stability	
		RSD (%)	Accuracy (%)	RSD (%)	Accuracy (%)	RSD (%)	Accuracy (%)
Scutellarin	0.8	1.85	98.0	2.64	95.4	2.40	97.1
	10	2.63	95.0	2.16	95.4	2.74	95.4
	60	2.07	102	2.53	103	2.44	103
Baicalin	4	1.39	97.6	1.71	99.1	1.90	97.9
	50	2.89	95.6	0.96	98.9	2.44	96.7
	300	1.43	95.5	2.16	95.4	1.74	95.1
Chrysin-7-O-glucuronide	0.8	1.36	104	2.17	104	2.06	104
	10	0.84	105	2.10	105	2.25	105
	60	2.24	95.6	2.84	104	2.70	102
Wogonoside	1.6	0.27	101	1.32	101	2.15	101
	20	0.55	104	1.09	105	1.25	105
	120	0.58	101	2.03	100	2.02	101
Baicalein	1.6	0.33	100	0.47	99.4	0.43	100
	20	2.65	100	2.19	103	2.70	101
	120	1.18	105	2.26	105	1.98	105
Wogonin	0.8	0.17	95	1.40	95	1.42	95.5
	10	2.62	103	1.20	105	2.69	104
	60	1.17	105	2.23	104	1.22	105
Chrysin	0.8	0.58	110	2.29	110	2.79	109
	10	0.53	100	1.58	99.7	2.37	99.1
	60	0.53	100	2.18	99.7	1.91	99.1
Oroxylin A	0.8	1.58	95.0	2.32	92.6	2.26	94.9
	10	1.96	107	2.07	108	1.94	107
	60	1.16	107	2.48	106	1.61	107

**Table 4:** The recovery of the eight bioactive compounds (n = 3).

Analyte	Original (μg)	Spiked (μg)	Found (μg)	Average recovery (%)	RSD (%)
Scutellarin	2.38	2.30	4.72	102	2.24
Baicalin	481	415	891	98.8	2.69
Chrysin-7-O-glucuronide	9.12	9.00	18.3	103	1.39
Wogonoside	125	102	229	101	2.97
Baicalein	20.8	18.0	39.4	103	2.35
Wogonin	8.65	8.10	17.1	104	1.53
Chrysin	1.62	1.40	3.00	98.7	2.79
Oroxylin A	2.43	1.92	3.63	58.9	1.84

### NA inhibitory activity integration of chromatography

**Fig 3:**
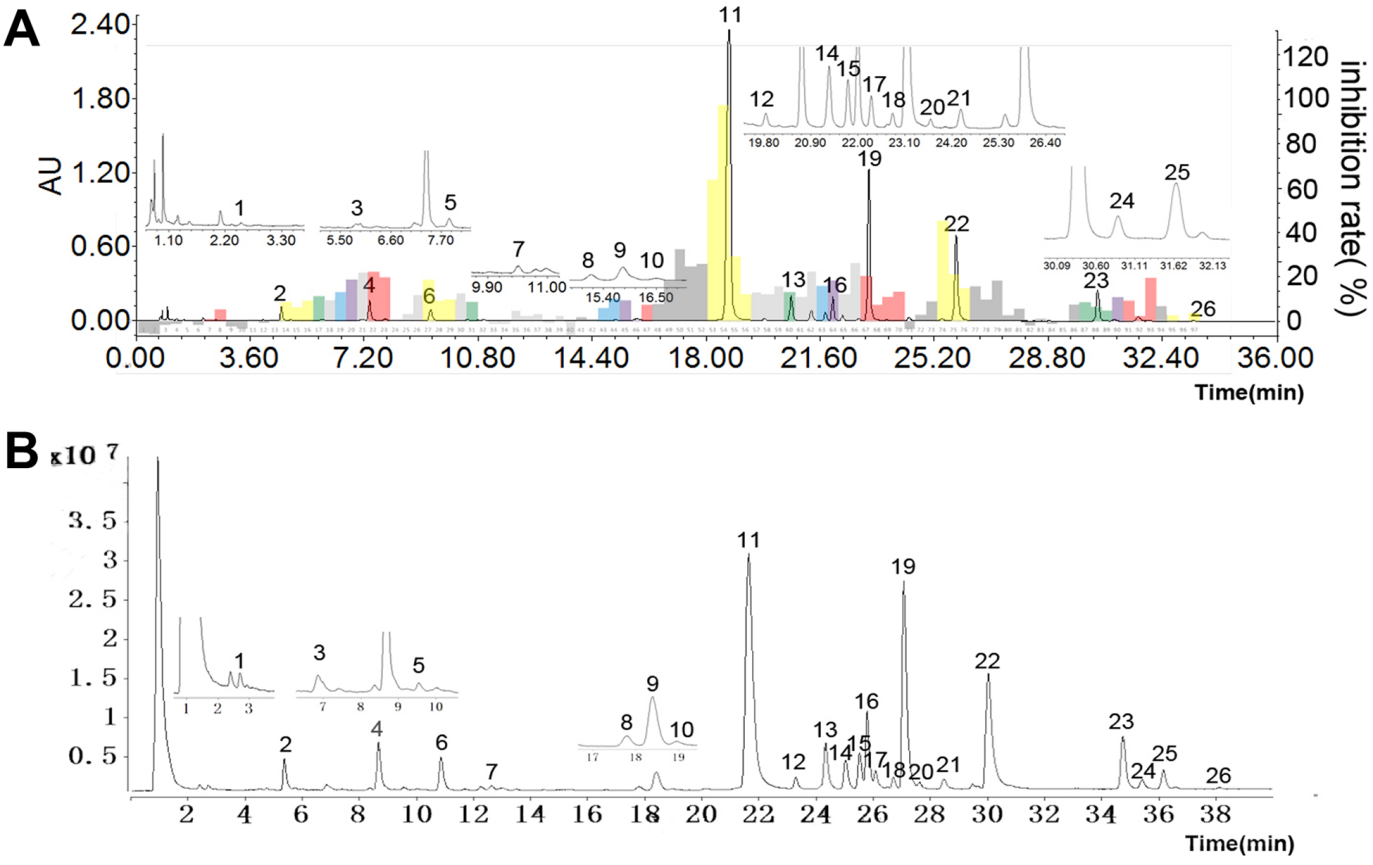
Chromatogram of activity-integrated fingerprints of RS extracts. **Chromatography (A) and total ion chromatography (B) of 26 compounds with NA inhibitory activity.** Colored bar means the fraction collection purity > 98%.

To integrate the compound concentration and bioactivity from the RS extract, the UPLC-DAD-FC system was used to separate, quantify, collect and enrich the compounds. One RS sample (labeled as S3 in Table 1) was analyzed by this method. 26 peaks of compounds in RS extract were detected by DAD detector. The fraction collector was set up at an interval time of 21s for the purity of most of the fractions. To make sure get enough amounts of components with low concentration in RS extract, enriching the samples of 10, 30, 50, 70 and 100 times were tested. The result shows for 10 times and 30 times enriching collection, some fractions were still undetectable for the NA inhibitory activity. We found that 50 times was the minimal enriching requirement for all fractions bioactivity to be detectable. The total 100 fraction were assayed for NA inhibitory activity, which was reflected with the inhibition rate (%) (Fig 3A). By integrating fraction bioactivity with chromatography analysis data, 26 components in Radix *Scutellariae* extracts were shown the neuraminidase inhibitory activity.

To searching for compounds with the highest potency of NA inhibitory activity, we integrated the NA inhibitory activity data with the chromatography quantity data. Interestingly, we found several components were identified to show relative high NA inhibitory activity. For example, the component (3’,5,5’,7-Tetrahydroxy-2’,8-dimethoxyflavone, Peak 9) which is at low concentration detected in RS extract exhibited comparable high NA inhibitory activity. The components with low concentration but high NA inhibitory activity will be further studied in our future work.

The quantification of component concentration and their NA inhibitory activity in this method could be finished at one step. The most current methods have to do this work at two individual steps, which takes time of months, but our method only takes 3 days for completion. Another advantage of this method was that a very little amount of herbal RS, such as 1 g, is sufficient for analysis, while several kilo grams of herbal RS is required in most other methods for detectable results and analysis. Moreover, in this method, the total of 26 compounds were identified at one time running, which is much more efficient than other methods that only detected several compounds at one time running [21].

### Qualitative analysis of 26 compounds in Radix *Scutellariae* extract

The UPLC-Q-TOF-MS system was used to identify 26 activity compounds in RS. The total ion chromatography showed 26 peaks totally in RS extract (Fig 3B). As shown in Table 5, chromatography peak **5, 10, 11, 15, 19, 22, 23, 24, 25** were identified to be scutellarin, scutellarein, baicalin, chrysin-7-O-glucuronide, wogonoside, baicalein, wogonin, chrysin and oroxylin A, respectively, by comparing their retention time (Rt), UV data and Q-Tof data with different standards, respectively [15,22]. Peak **1, 7, 9, 20** was identified to be darendoside A, 8-Arabinosyl-6-glucosyl-2’, 3, 5, 7-dihydroxyflavone and 3’, 5, 5’, 7-Tetrahydroxy-2’, 8-dimethoxyflavone and 5, 7-Dihydroxy-6, 8-dimethoxy flavone-7-O-glucuronide, respectively, based on previous studies [22]. Peaks **2 and 8** exhibited typical UV absorbance of flavanones band II situated at a region of long wavelength (more than 280 nm). On the other hand, band I presents with no signal or is only detected as a low signal. The negative ESI analysis of peak **2** gave the [M−H]^−^ ion at m/z 303, with characteristic fragment ions as m/z 217 [M−H−5OH]^−^. Negative ESI analysis of **8** gave the [M−H]^−^ ion at m/z 287, with peaks of m/z 161 [C_9_H_6_O_3_−H]^−^, m/z 125 [C_6_H_6_O_3_−H]^−^ [23]. **Peak 2, 8** were identified as 2’, 3, 4’, 5, 7-Pentahydroxyflavanone and 4’, 5, 7, 8-Tetrahydroxyflavanone, respectively. Peaks **4** and **6** showed same [M−H]^−^ ions at *m*/*z* 547. Data from the UV demonstrated maximum absorption wavelength at 272 and 315 nm. Their AUTO/MS fragments gave ions of [M−H−60]^−^ at *m*/*z* 487, [M−H−90]^−^ at *m*/z457 and [M−H−120]^−^ at *m*/z 427 respectively, comparable to the characteristic fragment ions of peaks 4 identified as C-glycoside [24]. The UV spectrum also showed different retention time by C18 reversed-phase column [23]. Thus, peak **4** and **6** were initially identified as chrysin-6-C-ara-8-C-glu and chrysin-6-C-glu-8-C-ara, respectively. Peak **12** gave [M−H]^−^ ion at m/z 447. In Auto/MS mode, it produced an ion at m/z 271 [M−H−176]^−^, inferring it presents with a glucuronic acid. And it gave ions of [M−H−176−18]^−^ at m/z 253, due to the loss of H_2_O group, suggesting the presence of two −OH in or the positions. Peak **12** was identified as 5,6-Dihydroxy-7-O-glu acid flavanone[24]. Negative ESI analysis of Peak **13** gave the [M−H]^−^ ion at *m*/*z* 445 and the aglycone anion at *m*/*z* 270. Furthermore, the same fragmentation patterns were observed as was for the Auto/MS of the protonated norwogonin, thus identifying peak **13** as a glucuronide of norwogonin. Also, spectrum from the UV showed the maximum absorption wavelength at 231nm and 280 nm and it was similar to that of norwogonin, showing the glycosylation of 7 −OH for **13**. Thus, peak **13** was positively identified as norwogonin-7-O-glucuronide [22].

**Table 5:** The qualitative information of Radix *scutellariae*.

No.	Retention time	Selected ion	Formula	Ppm	UVλ_max_	ELS-MS	ELS-MS/MS	Identification results
1	2.60	[M-H]^-^	C_19_H_28_O_11_	2.96	234, 295	431.1546	299.1154, 233.0674, 191.0552 161.0447,149.0447, 131.0338	Darendoside A
2	5.386	[M-H]^-^	C_15_H_12_O_7_	1.76	231,290	303.0503	217.0506, 177.0190, 149.0247 125.0246	2’,3,4’,5,7-Pentahydroxyflavanone
3	6.879	[M-H]^-^	C_26_H_30_O_14_	4.69	240,295	565.1536	427.1022, 385.0919, 355.0819 329.0880, 299.0780,269.0673, 239.0562, 209.0458, 167.0355	/
4	8.622	[M-H]^-^	C_26_H_28_O_13_	3.28	272,315	547.1439	547.1438, 487.1227, 457.1117, 427.1019, 367.0815, 337.0708	Chrysin-6-C-ara-8-C-glu
5	9.494	[M-H]^-^	C_21_H_18_O_12_	1.3	280,335	461.0716	286.0434, 285.0387, 284.0240 283.0246, 255.0322,113.0236	Scutellarin
6	10.598	[M-H]^-^	C_26_H_28_O_13_	3.28	272,315	547.1451	457.1115, 427.1018,367.0800 337.0706	Chrysin-6-C-glu-8-C-ara
7	12.283	[M-H]^-^	C_27_H_34_O_14_	3.37	235,273	581.1861	563.1770, 491.1553, 462.1466 461.1442, 329.1018, 299.0920 167.0347	8-Arabinosyl-6-glucosyl-2’,3,5,7-dihydroxyflavone
8	17.572	[M-H]^-^	C_15_H_12_O_6_	0.62	230,288	287.0559	161.0236, 125.0239	4’, 5, 7, 8-Tetrahydroxyflavanone
9	18.269	[M-H]^-^	C_17_H_14_O_8_	1.12	230,264	345.0612	330.0374, 315.0137,	3’,5,5’,7-Tetrahydroxy-2’,8- dimethoxyflavone
10	18.734	[M-H]^-^	C_15_H_12_O_6_	3.07	230,290	287.0550	201.0550, 161.0246, 133.029 125.024	Scutellarein
11	21.598	[M-H]^-^	C_21_H_18_O_11_	2.34	276,315	445.0766	269.0456,239.0348,175.0271, 113.0245	Baicalin
12	23.150	[M-H]^-^	C_21_H_20_O_11_	0.21	231,290	447.0932	271.0601, 253.0656, 164.9822 113.0240	5,6-Dihydroxy-7-O-glu acid flavanone
13	24.254	[M-H]^-^	C_21_H_18_O_11_	-0.31	231,280	445.0777	270.0476, 269.0446, 267.0286 113.0240	Norwogonin-7-O-glucuronide
14	24.952	[M-H]^-^	C_22_H_20_O_12_	1.93	283,322	475.0873	299.0552, 284.0318, 113.024	5,7,8-Trihydroxy-6-methoxy flavone-7-O-glucuronide
15	25.475	[M-H]^-^	C_21_H_18_O_10_	2.68	267,304	429.0816	253.0504, 113.0243	Chrysin-7-O-glucuronide
16	25.824	[M-H]^-^	C_22_H_20_O_11_	3.66	272,312	459.0916	283.0610, 268.0376, 113.0244	Oroxylin A-7-O-glucuronidea
17	26.056	[M-H]^-^	C_22_H_20_O_12_	2.78	279,316	475.0872	299.056, 284.0326, 113.0245	5,6,7-Trihydroxy-8-methoxy flavone-7-O-glucuronideb
18	26.695	[M-H]^-^	C_21_H_18_O_11_	2.53	274,371	445.0765	269.0456, 113.0246	Baicalein-6-O-glucuronide
19	27.119	[M-H]^-^	C_22_H_20_O_11_	3.10	274,349	459.0919	283.0613, 268.0379,113.0249	Wogonoside
20	27.567	[M-H]^-^	C_23_H_22_O_12_	3.01	278,318	489.1028	313.0718, 298.0480, 283.0246 113.0246	5,7-Dihydroxy-6,8-dimethoxy flavone-7-O-glucuronide
21	28.380	[M-H]^-^	C_15_H_10_O_5_	0.05	279	269.0455	269.0454, 241.0497, 197.0604	Norwogonin
22	29.906	[M-H]^-^	C_15_H_10_O_5_	-1.72	276,323	269.0460	251.0342, 241.0495, 223.0393, 169.0655	Baicalein
23	34.715	[M-H]^-^	C_16_H_12_O_5_	-1.03	274	283.0612	268.0381, 163.0038	Wogonin
24	35.412	[M-H]^-^	C_15_H_10_O_4_	-2.27	268,313	253.0512	253.0502, 209.0605	Chrysin
25	36.186	[M-H]^+^	C_16_H_12_O_5_	-0.77	269	285.0755	285.0750,270.0518	Oroxylin A
25’	36.186	[M-H]^-^	C_19_H_18_O_8_	-2.14	272,313	373.1097	358.0835,343.0600,328.0491	Skullcapflavon II
26	37.635	[M-H]^-^	C_18_H_16_O_7_	0.31	277,336	343.0968	328.0720, 313.0488, 310.0607 298.0374	Tenaxin I

Peaks **14 and 17** exhibited the [M−H]^−^ ion at *m*/*z* 475 and the aglycone anion at *m*/*z* 299. Auto/MS of the two *m*/*z* 299 ions all produced the fragment of [M−H−CH_3_]^−^at *m*/*z* 284. This fragment shows the existence of a methoxyl and three hydroxyls in present on each aglycone of these two compounds. The maximum UV absorption wavelength from the UV data of these two flavonoids is very different. Both peak **14** and **17** showed a low-intensity although a clear absorption of band I was present at 322nm and 316 nm respectively, which is characteristic of flavones with 6-oxygenation. Peak **14** presented the band II absorption at 283 nm instead of that of peak **17** (279 nm). Thus, peak **14** was identified as 5,7,8-trihydroxy-6-methoxyflavone-7-O-glucuronide and peak **17** was think of 5,6,7-trihydroxy-8-methoxyflavone-7-O-glucuronide [22].

Peak **16** and **19** represent a pair of isomers. Both have fragments giving a [M−H]^−^ at m/z 459 and m/z 459 gave the ion of [M−H−176]^−^ at m/z 283, which involves the loss of a glucuronic acid, then m/z 283 produced ion of [M−H−176−15]^−^ at m/z 268, which suggests the presence of a −CH_3_ group. From previous studies on flavonoids in RS, peak **19** was named as wogonoside by comparing with a standard based on the retention time in a previously reported HPLC method [24]. Peak **16** was identified as oroxylin A-7-O-glucuronide.

Peak **21** and **22** both had the [M−H] ^−^ ion at m/z 269. In comparison with known standards, peak **22** was identified as baicalein. In Auto/MS data, the fragmentation of **21** was very similar to that of **22**. By screening the known flavones with maximum absorption wavelength of 279nm in RS, peak **21** was identified as norwogonin [24].

Peak **25** and **25’** have same retention time. Peak **25’**produced the [M−H] ^−^ ion at m/z 373. Its Auto/MS produced ions at 358 [M−H−CH_3_] ^−^, 343 [M−H−2CH_3_] ^−^, 328 [M−H−3CH_3_] ^−^, These ions should be derived from four −OCH_3_ groups which are located on the flavone skeleton. By searching the known flavones in RS, it was named as skullcapflavone II, tentatively [24].

Peak **26** produced the [M−H] ^−^ ion at m/z 343. Analyzing their fragments, an inference was made that both of them have three—OCH3 groups from observing the ions with m/z 328, m/z 313 and m/z298. Based on these results and previous knowledge on the flavones in RS, peak **26** was named tenaxin I [24]. All results were shown in Table 4.

This method combines UPLC-UV-FC and UPLC-Q-TOF-MS to assess both quantity and quality of components, as well as the corresponding bioactivity (Fig 3). From this result the highest potent NA inhibitors were identified, such as baicalin, wogonoside, etc.

### Verification of the NA inhibitory activity of compounds in Radix *Scutellariae*

**Fig 4:**
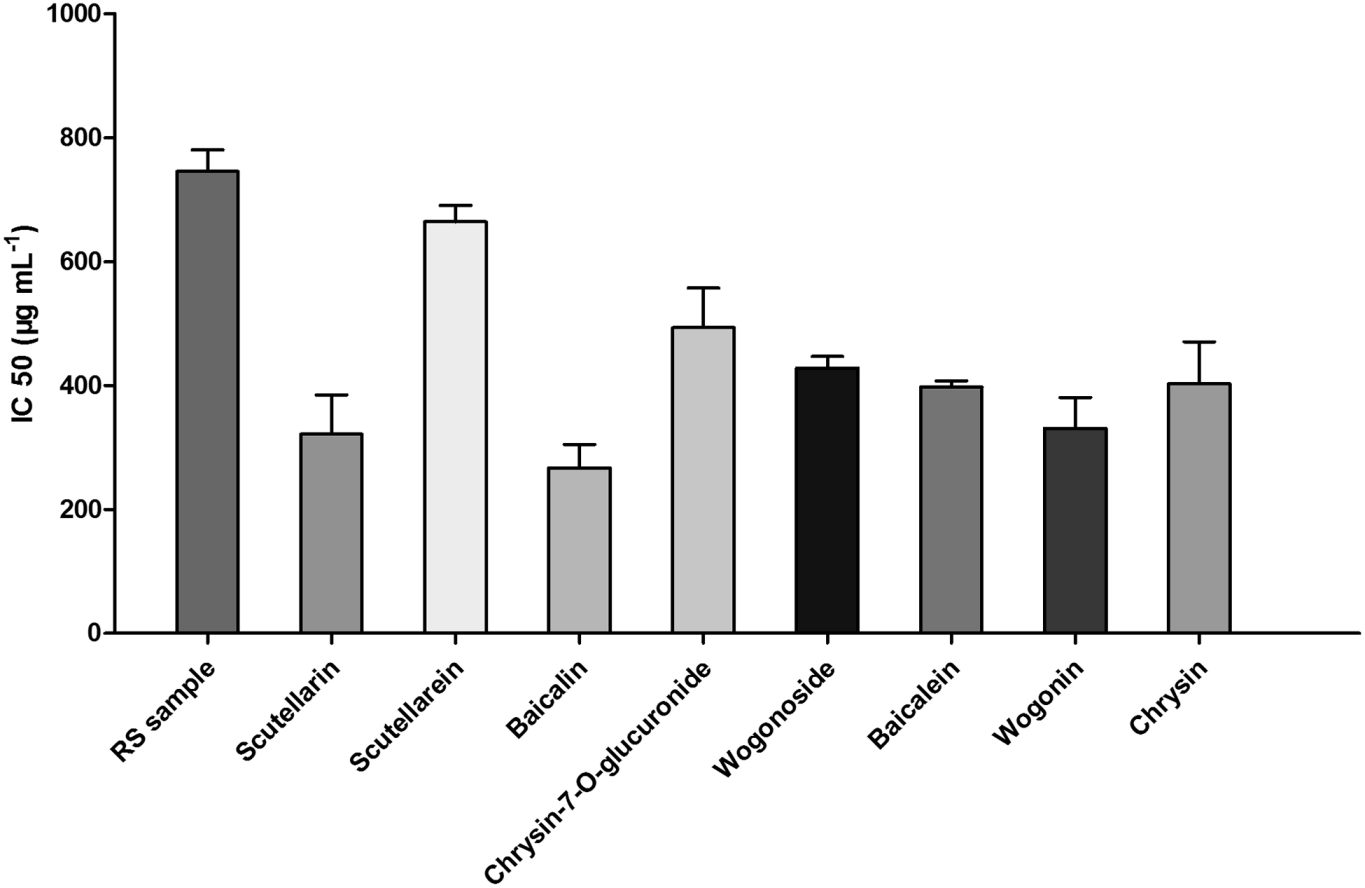
The NA inhibitory activity of compounds isolated from RS. The IC_50_ of Radix *scutellariae* extract scutellarin, scutellarein, baicalin, baicalein, wogonoside, wogonin, chrysin-7-O-glucuronideand chrysin by an NA inhibitory screening kit.

To confirm the activity-integrated method, inhibitory activity assays of the RS extracts and the 9 bioactive standards (purity > 98%) were performed. The IC_50_ value of the RS extract was 745.5 ± 35.2 μg mL^-1^. For the 9 standards the IC50 values were 321.8 ± 62.8 μg mL^-1^ for scutellarin, 664.5 ± 26.2 μg mL^-1^for scutellarein, 267.3 ± 37.8 μg mL^-1^ for baicalin, 493.6 ± 64.2 μg mL^-1^ for chrysin-7-O-glucuronide, 428.3 ± 18.9 μg mL^-1^ for wogonoside, 397.7 ± 9.91 μg mL^-1^ for baicalein, 331.1 ± 49.4 μg mL^-1^ for wogonin, 402.80 ± 67.8 μg mL^-1^ for chrysin and the more than 1000 μg·mL^-1^ for oroxylin A (Fig 4). Specifically, baicalin, baicalein, wogonoside and wogonin showed the high NA inhibitory activity, with the baicalin showing the highest NA inhibitory activity (Fig 4). All these compounds were shown high concentration in RS, and indicated their high potential in pharmacological values. Oroxylin A has the very weak NA inhibitory activity with the IC_50_ of more than 1000 μg·mL^-1^, which is different from the result of UPLC-UV-FC with oroxylin A showing the relative high NA inhibitory activity (Fig 3). The possible reason is that the skullcapflavone II, which override with oroxylin A in the same peak with mass spectrometry assay, was mixed in one fraction.

**Fig 5:**
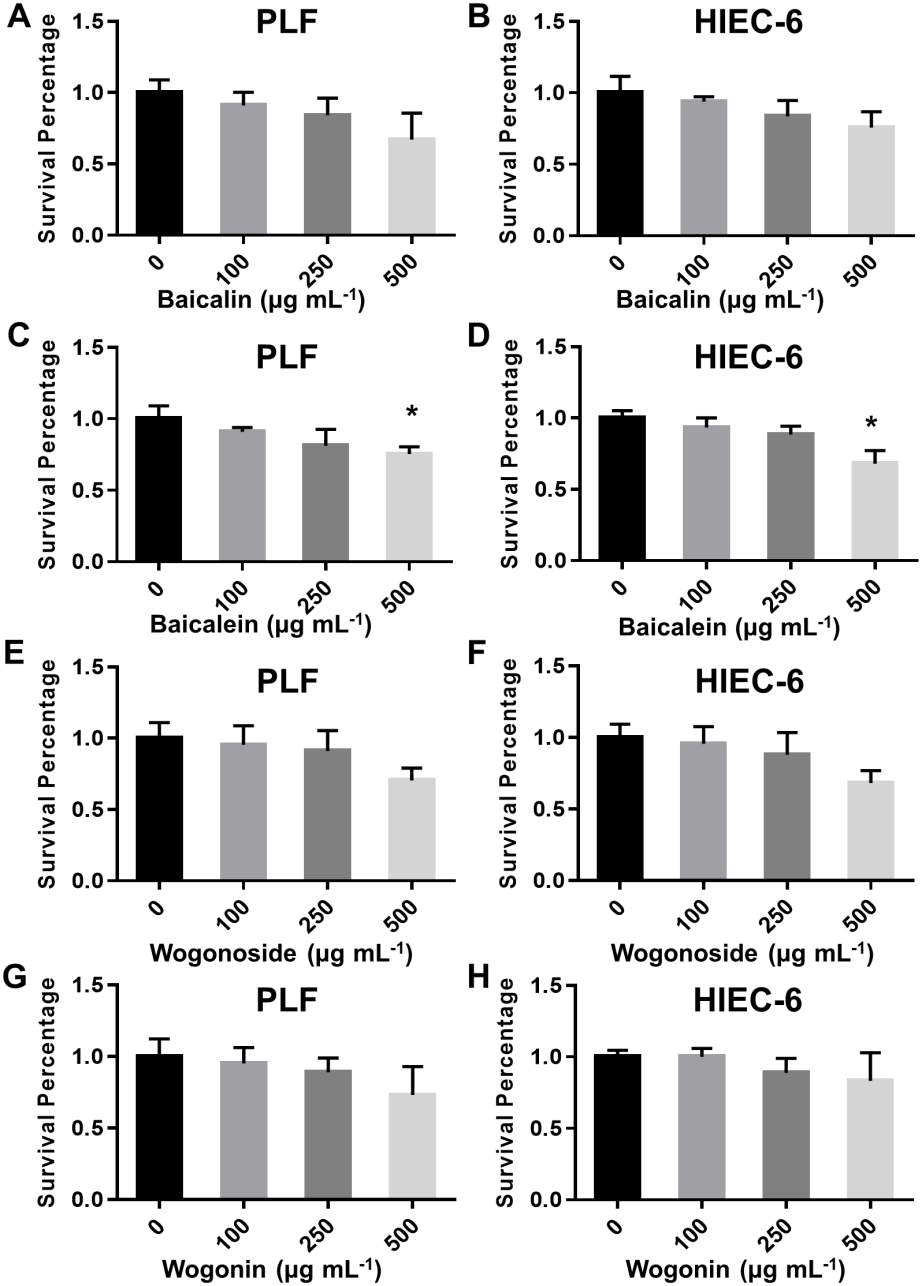
The cellular toxicity of baicalin, baicalein, wogonoside and wogonin. PLF and HIEC-6 cells were treated with indicated drugs with different concentrations as indicated for 24 hours and collected to evaluate cellular viability.

**Fig 6:**
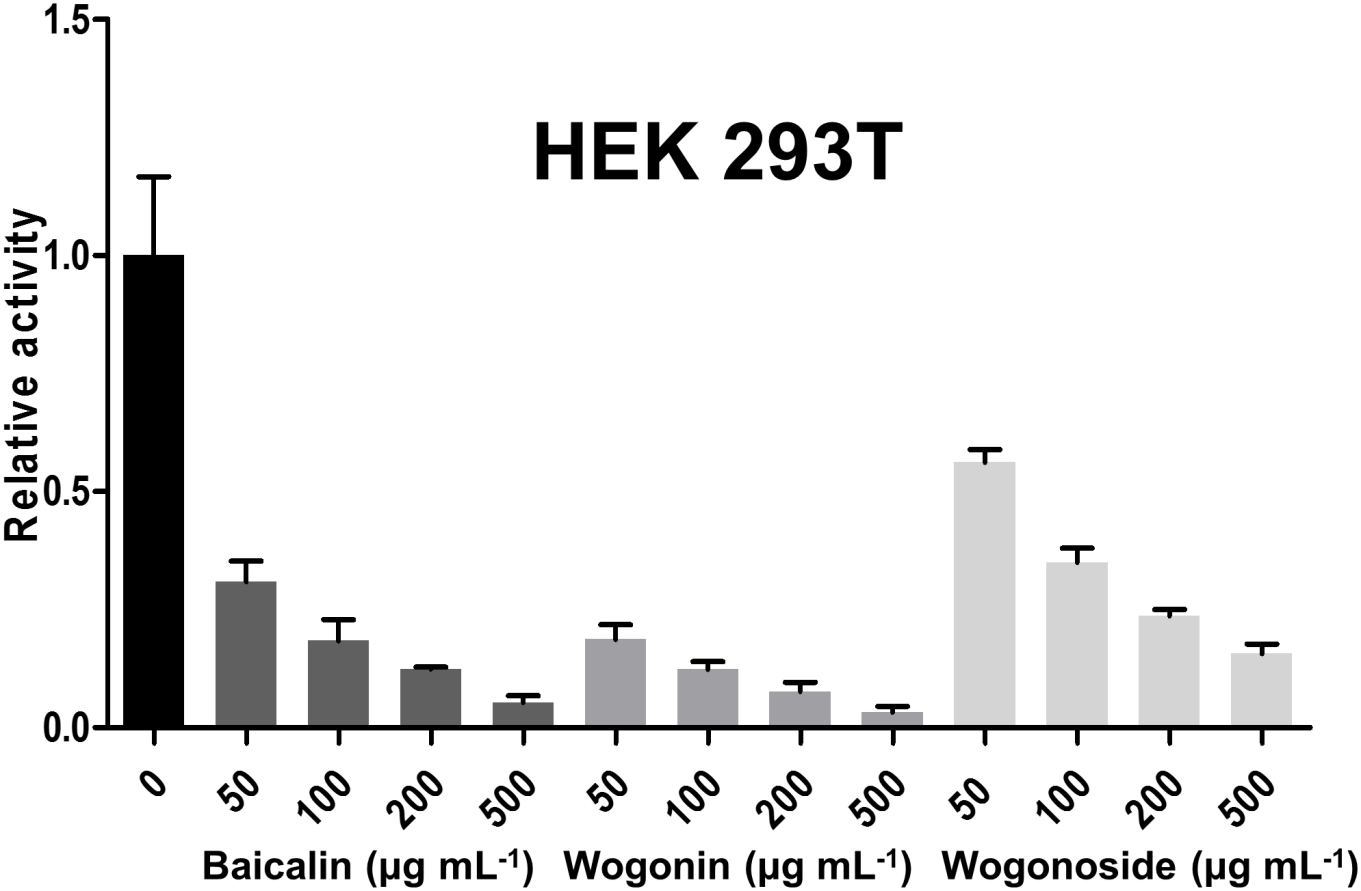
The relative NA inhibitory activity of baicalin, wogonin and wogonoside on HEK293Tcells.

To assay the impacts of the components on cellular activity, the cell survival rate was detected by using both primary lung (PLF) and intestine (HIEC-6) cells lines treated with indicated components at corresponding IC_50_ concentration. The result showed that baicalin, wogonoside and wogonin did not have significant effect on these cell survivals (Fig 5). To further confirm the bioactivity of the component, we treated HEK293T cells ectopically expressing viral neuraminidase with the indicated component of baicalin, wogonoside and wogonin at conditions of 50, 100, 200 and 500 μg mL^-1^. The whole cell lysate was extracted for NA activity assay. Baicalin and wogonin were shown the high inhibition on NA in the cells at all tested concentrations (Fig 6). This result is in agreement with the previous data (Fig 4). Our method identified the major component of flavones in RS extract showing high NA inhibitory activity, suggesting the potential biomedical value of herbal medicine.

## Quantitative analysis of the different types of Radix *Scutellariae* by 26 NA inhibitory activity compounds

To determine the contents of each bioactive component in NA inhibitory of 18 RS samples, the UPLC-DAD was used. There are 18 RS samples which include three group of 1. biennially cultivated RS, 2. perennially (3 years or more) cultivated RS and 3. wild RS. The detailed information of the 18 RS samples was summarized in Table 1. Concentrations of the 26 components in 18 RS samples were shown in Table 6. Seven of active components were quantified with standards, including scutellarin, baicalin, chrysin-7-O-glucuronide, wogonoside. baicalein, wogonin and chrysin. Specifically, the corresponding concentration were 0.44–0.54 μg mg^-1^, 244.23–421.18 μg mg^-1^, 4.72–10.32 μg mg^-1^, 52.91–104.89 μg mg^-1^, 8.21–32.23 μg mg^-1^ and 1.60–2.73 mg mg^−1^, respectively. The other 19 components were semi-quantitatively determined by the calibration curve of scutellarin due to lacking the corresponding standard.

**Table 6:** The quantitative results of Radix *scutellariae*.

	Content of compound(mg/g)																									
sample	1	2	3	4	5	6	7	8	9	10	11	12	13	14	15	16	17	18	19	20	21	22	23	24	25	26.
S1	0.00	1.24	0.13	2.32	0.19	1.64	0.04	0.07	0.52	0.00	59.99	0.20	2.76	1.04	1.14	2.63	0.41	0.04	15.57	0.00	0.00	2.37	1.33	0.45	0.53	0.00
S2	0.00	1.96	0.00	2.93	0.18	1.78	0.22	0.16	0.54	0.00	67.95	0.48	3.99	0.97	1.71	3.63	0.77	0.37	17.71	0.15	0.37	5.30	2.30	0.41	1.35	0.02
S3	0.00	1.70	0.00	3.32	0.61	2.10	0.40	0.42	0.26	0.00	68.01	1.47	7.51	1.61	1.49	6.59	0.76	0.00	15.84	0.18	0.64	4.48	1.98	0.51	1.61	0.00
S4	0.00	1.53	0.19	3.39	0.69	1.87	0.47	0.25	0.29	0.00	73.98	1.02	6.22	2.04	1.55	6.66	0.72	0.09	17.38	0.63	0.22	3.48	1.52	0.41	1.73	0.00
S5	0.10	1.69	0.08	3.88	1.11	2.40	0.58	0.17	0.87	0.00	73.30	1.24	5.85	1.54	1.72	13.2	1.39	0.49	14.86	0.90	0.61	3.89	1.41	0.50	1.95	0.00
S6	0.00	1.55	0.07	3.35	0.24	2.46	0.35	0.05	0.07	0.00	71.01	1.47	7.30	1.49	1.90	7.23	0.86	0.11	18.84	0.61	0.41	2.27	1.13	0.45	1.06	0.00
S7	0.18	1.58	0.10	3.73	0.37	2.61	0.31	0.28	0.25	0.00	81.12	1.77	7.33	1.83	2.06	7.84	0.63	0.08	19.24	0.68	0.43	2.99	1.15	0.49	1.23	0.00
S8	0.02	1.56	0.00	3.26	0.77	2.02	0.36	0.26	0.28	0.00	70.24	0.71	5.55	0.99	1.91	9.11	0.92	0.14	15.49	0.54	0.52	3.73	1.50	0.53	1.66	0.00
S9	0.00	1.15	0.06	2.73	0.25	1.87	0.27	0.03	0.27	0.00	69.52	1.07	3.87	1.32	1.21	0.88	0.84	0.12	13.74	0.37	0.11	1.64	0.75	0.33	0.95	0.00
S10	0.01	0.63	0.04	3.28	0.59	2.12	0.38	0.11	0.40	0.00	69.62	0.43	4.71	1.97	0.94	4.00	0.14	0.04	15.87	0.57	0.47	4.57	1.41	0.53	1.37	0.00
S11	0.04	1.18	0.17	2.61	0.24	1.65	0.09	0.09	0.32	0.00	71.24	0.65	3.95	1.64	1.26	4.75	0.26	0.28	18.33	0.15	0.47	3.68	2.11	0.43	1.07	0.00
S12	0.01	1.90	0.10	2.59	0.09	1.69	0.18	0.29	0.68	0.00	53.70	0.31	2.98	1.15	1.32	1.77	0.34	0.07	14.02	0.09	0.74	6.45	2.69	0.50	1.21	0.08
S13	0.04	1.01	0.01	4.14	0.72	2.81	0.41	0.04	0.38	0.00	84.24	0.62	5.80	2.33	1.35	5.64	0.25	0.00	20.98	0.69	0.32	3.50	1.17	0.46	1.23	0.00
S14	0.00	1.02	0.06	3.02	0.26	2.04	0.33	0.12	0.44	0.00	64.48	0.44	4.38	1.53	1.11	3.87	0.30	0.02	17.06	0.35	0.35	3.80	1.31	0.42	1.46	0.00
S15	0.06	2.82	0.00	3.38	0.32	2.28	0.51	0.41	0.63	0.00	70.99	0.84	3.70	0.98	1.69	7.00	1.32	0.63	10.58	0.16	0.13	5.18	1.52	0.32	2.66	0.08
S16	0.06	2.82	0.00	2.79	0.14	1.81	0.31	0.11	0.72	0.00	66.56	1.64	5.46	1.13	1.31	7.02	2.26	0.63	14.97	0.36	0.19	4.94	1.41	0.36	2.35	0.00
S17	0.00	2.23	0.00	2.37	0.29	1.36	0.23	0.36	0.05	0.00	48.85	2.11	4.10	0.72	1.43	8.31	1.26	0.26	11.68	0.54	0.26	3.12	1.72	0.55	3.50	0.19
S18	0.18	4.76	0.03	2.77	0.61	1.92	0.29	0.21	8.72	0.24	49.77	1.80	1.70	0.70	1.45	13.38	0.06	0.07	15.23	0.25	0.18	4.56	1.18	0.49	2.48	0.00

The flavones (baicalin and wogonoside) are the most abundant component in RS extract among all the 18 samples with an average concentration of 116.54 mg g^-1^. It is also found that the concentration of flavones (baicalin and wogonoside) in perennially (3 years or more) cultivated RS was significantly higher than that in biennially cultivated RS. Baicalin is the major component in total flavones, which reaches up to more than 5% of the gross total weight of RS sample Table 6, So that baicalin was expected to serve as the major contributor to NA inhibitory activity of RS extract.

### Hierarchical cluster analysis of the different types of Radix Scutellariae by 26 NA inhibitory activity compounds

**Fig 7:**
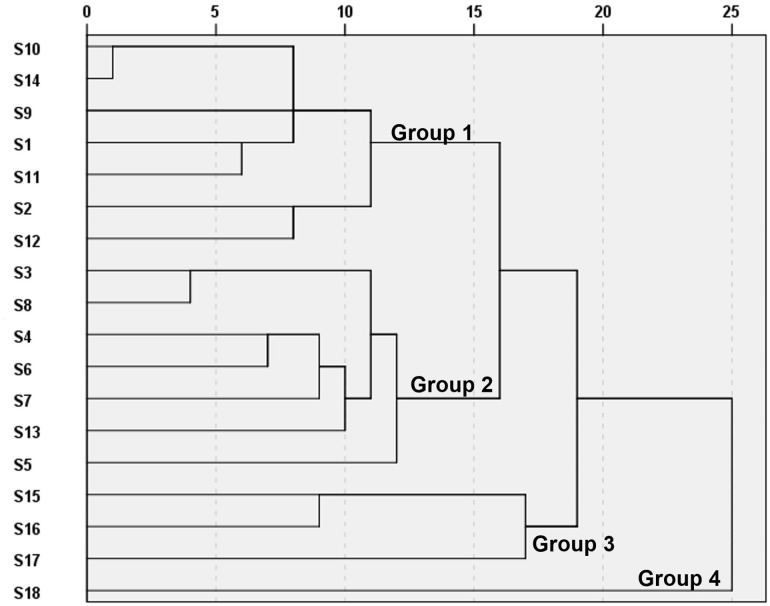
HCA dendrogram of 18 RS samples based on the NA inhibitory activity of compounds. Biennially cultivated RS (Group 1), perennially cultivated RS (Group 2), wild RS (Group 3) and plateau area RS (Group 4).

Hierarchical cluster analysis (HCA) is defined as a statistical method to analyze and cluster data by measuring the similarity in samples. HCA is preferential to cluster objects with highest similarities into one group. HCA was also applied to distinguish the different HM samples [25]. The 18 RS samples were assayed with HCA according their concentration of 26 compounds screened with UPLC/Q-TOF-MS-FC (Fig 3) HCA dendrogram (Fig 7) showed that 18 RS samples were classified into 4 groups based on concentration and NA inhibitory bioactivity of each sample (Fig 3, Table 6). The first group (group I) contains the most biennially cultivated RS, including S10, S14, S9, S1, S11, S2, and S12. Moreover, the second group (group II) contains the perennially (3 years or more) cultivated RS, inducing S3, S8, S6, S7, S4, S5 and S13. This result demonstrated that component concentration and NA inhibitory bioactivity in RS extract were distinct between biennially cultivated and the perennially (3 years or more) cultivated RS. Group III consisted of the three wild RS (S15, S16 and S17) samples. It implied that the effects of wildness on RS contents correlated with the age of RS. The sample S18, from plateau Area, was clustered into group IV individually. These results suggest that high altitude climate may have a special role on the chemical contents of RS. From HCA methods, it was indicated that the influence of origins on the quality of RS was more than that of cultivated time.

## Conclusions

A new integrated UPLC-DAD-FC/UPLC-Q-TOF-MS method has been developed and successfully applied in screening natural neuraminidase inhibitors in RS extract. Using our new developed analysis methods, we found the main neuraminidase inhibitors in RS extract were baicalin, baicalein, wogonoside and wogonin. All of them have indicated the treatment effect in preventing influenza. The treatment effects of RS contents were more significantly detected in old RS than in the young one. Besides the concentration and components of the neuraminidase inhibitors in RS extract, the NA inhibitory activities of each component were also assayed by our fast method. Compared with traditional chromatographic separation and inhibitory activity methods, our neuraminidase inhibitory activity integrated UPLC-DAD-FC/UPLC-Q-TOF-MS method was more efficient, economic and fast in screening and identifying anti-viral components from heat-clearing drugs in herbal medicines.

## References

[1] Zhu HY, Han L, Shi XL, Wang BL, Huang H, Wang X, et al (2015) Baicalin inhibits autophagy induced by influenza A virus H3N2. Antiviral Res 113: 62–70. 10.1016/j.antiviral.2014.11.003 25446340

[2] Rakers C, Schwerdtfeger SM, Mortier J, Duwe S, Wolff T, Wolber G, et al (2014) Inhibitory potency of flavonoid derivatives on influenza virus neuraminidase. Bioorg Med Chem Lett 24: 4312–4317. 10.1016/j.bmcl.2014.07.010 25096296

[3] von Itzstein M (2007) The war against influenza: discovery and development of sialidase inhibitors. Nat Rev Drug Discov 6: 967–974. 10.1038/nrd2400 18049471

[4] Nakano T, Shiosakai K (2014) Spread of viral infection to family members from influenza patients treated with a neuraminidase inhibitor. J Infect Chemother 20: 401–406. 10.1016/j.jiac.2014.01.012 24787736

[5] Abed Y, Pizzorno A, Bouhy X, Boivin G (2015) Permissive changes in the neuraminidase play a dominant role in improving the viral fitness of oseltamivir-resistant seasonal influenza A(H1N1) strains. Antiviral Res 114: 57–61. 10.1016/j.antiviral.2014.12.006 25512229

[6] Samson M, Pizzorno A, Abed Y, Boivin G (2013) Influenza virus resistance to neuraminidase inhibitors. Antiviral Res 98: 174–185. 10.1016/j.antiviral.2013.03.014 23523943

[7] Huang SF, Fung CP, Perng DW, Wang FD (2015) Effects of corticosteroid and neuraminidase inhibitors on survival in patients with respiratory distress induced by influenza virus. J Microbiol Immunol Infect. 10.1016/j.jmii.2015.08.016 26542650

[8] Tian L, Wang Z, Wu H, Wang S, Wang Y, Wang Y, et al (2011) Evaluation of the anti-neuraminidase activity of the traditional Chinese medicines and determination of the anti-influenza A virus effects of the neuraminidase inhibitory TCMs in vitro and in vivo. J Ethnopharmacol 137: 534–542. 10.1016/j.jep.2011.06.002 21699971

[9] He H, Han S, Zhang T, Zhang J, Wang S, Hou J (2012) Screening active compounds acting on the epidermal growth factor receptor from Radix scutellariae via cell membrane chromatography online coupled with HPLC/MS. J Pharm Biomed Anal 62: 196–202. 10.1016/j.jpba.2011.12.025 22260968

[10] Cuong TD, Hung TM, Lee JS, Weon KY, Woo MH, Min BS (2015) Anti-inflammatory activity of phenolic compounds from the whole plant of Scutellaria indica. Bioorg Med Chem Lett 25: 1129–1134. 10.1016/j.bmcl.2014.12.055 25637363

[11] Lohani M, Ahuja M, Buabeid MA, Dean S, Dennis S, Suppiramaniam V, et al (2013) Anti-oxidative and DNA protecting effects of flavonoids-rich Scutellaria lateriflora. Nat Prod Commun 8: 1415–1418. 24354189

[12] Zhu B, Davie JK (2015) New insights into signalling-pathway alterations in rhabdomyosarcoma. Br J Cancer 112: 227–231. 10.1038/bjc.2014.471 25211658 PMC4453439

[13] Dai ZJ, Lu WF, Gao J, Kang HF, Ma YG, Zhang SQ, et al (2013) Anti-angiogenic effect of the total flavonoids in Scutellaria barbata D. Don. BMC Complement Altern Med 13: 150 10.1186/1472-6882-13-150 23815868 PMC3717098

[14] Guo SS, Shi YJ, Gao YJ, Su D, Cui XL (2009) The cytology mechanism of anti-parainfluenza virus infection of total flavone of Scutellaria barbata. Yao Xue Xue Bao 44: 1348–1352. 21351467

[15] Wang H, Cao J, Xu S, Gu D, Wang Y, Xiao S (2013) Depletion of high-abundance flavonoids by metal complexation and identification of low-abundance flavonoids in Scutellaria baicalensis Georgi. J Chromatogr A 1315: 107–117. 10.1016/j.chroma.2013.09.052 24075012

[16] Hour M-J, Huang S-H, Chang C-Y, Lin Y-K, Wang C-Y, Chang Y-S, et al (2013) Baicalein, Ethyl Acetate, and Chloroform Extracts of Scutellaria baicalensis Inhibit the Neuraminidase Activity of Pandemic 2009 H1N1 and Seasonal Influenza A Viruses. Evidence-Based Complementary and Alternative Medicine 2013: 1–11. 10.1155/2013/750803 PMC3705751 23864896

[17] Lu X, Liu F, Zeng H, Sheu T, Achenbach JE, Veguilla V, et al (2014) Evaluation of the antigenic relatedness and cross-protective immunity of the neuraminidase between human influenza A (H1N1) virus and highly pathogenic avian influenza A (H5N1) virus. Virology 454–455: 169–175. 24725943 10.1016/j.virol.2014.02.011

[18] Zhu B, Zhang M, Williams EM, Keller C, Mansoor A, Davie JK (2016) TBX2 represses PTEN in rhabdomyosarcoma and skeletal muscle. Oncogene 35: 4212–4224. 10.1038/onc.2015.486 26686089 PMC4916052

[19] Zhang M, Zhu B, Davie J (2015) Alternative splicing of MEF2C pre-mRNA controls its activity in normal myogenesis and promotes tumorigenicity in rhabdomyosarcoma cells. J Biol Chem 290: 310–324. 10.1074/jbc.M114.606277 25404735 PMC4281734

[20] Patel M, Giddings AM, Sechelski J, Olsen JC (2013) High efficiency gene transfer to airways of mice using influenza hemagglutinin pseudotyped lentiviral vectors. J Gene Med 15: 51–62. 10.1002/jgm.2695 23319179 PMC5300025

[21] Apea-Bah FB, Hanafi M, Dewi RT, Fajriah S, Darwaman A, Artanti N, et al (2009) Assessment of the DPPH and alpha-glucosidase inhibitory potential of gambier and qualitative identification of major bioactive compound. Journal of Medicinal Plants Research 3: 736–757.

[22] Liu G, Ma J, Chen Y, Tian Q, Shen Y, Wang X, et al (2009) Investigation of flavonoid profile of Scutellaria bacalensis Georgi by high performance liquid chromatography with diode array detection and electrospray ion trap mass spectrometry. J Chromatogr A 1216: 4809–4814. 10.1016/j.chroma.2009.04.021 19411078

[23] Seo ON, Kim G-S, Kim Y-H, Park S, Jeong SW, Lee SJ, et al (2013) Determination of polyphenol components of Korean Scutellaria baicalensis Georgi using liquid chromatography—tandem mass spectrometry: Contribution to overall antioxidant activity. Journal of Functional Foods 5: 1741–1750.

[24] Han J, Ye M, Xu M, Sun J, Wang B, Guo D (2007) Characterization of flavonoids in the traditional Chinese herbal medicine-Huangqin by liquid chromatography coupled with electrospray ionization mass spectrometry. J Chromatogr B Analyt Technol Biomed Life Sci 848: 355–362. 10.1016/j.jchromb.2006.10.061 17118721

[25] Yi T, Zhu L, Peng W-L, He X-C, Chen H-L, Li J, et al (2015) Comparison of ten major constituents in seven types of processed tea using HPLC-DAD-MS followed by principal component and hierarchical cluster analysis. LWT—Food Science and Technology 62: 194–201.

